# Collecting Duct Carcinomas Represent a Unique Tumor Entity Based on Genetic Alterations

**DOI:** 10.1371/journal.pone.0078137

**Published:** 2013-10-22

**Authors:** Frank Becker, Kerstin Junker, Martin Parr, Arndt Hartmann, Susanne Füssel, Marieta Toma, Rainer Grobholz, Thomas Pflugmann, Bernd Wullich, Arne Strauss, Carl Ludwig Behnes, Wolfgang Otto, Michael Stöckle, Volker Jung

**Affiliations:** 1 Department of Urology, University of the Saarland, Homburg/Saar, Germany; 2 Department of Pathology, University Hospital Erlangen, Erlangen, Germany; 3 Department of Urology, Technical University of Dresden, Dresden, Germany; 4 Department of Pathology, Technical University of Dresden, Dresden, Germany; 5 Department of Pathology, Kantonsspital Aarau, Aarau, Switzerland; 6 Department of Urology, St. Franziskus Clinics, Mönchengladbach, Germany; 7 Department of Urology, University Hospital Erlangen, Erlangen, Germany; 8 Department of Urology, Georg-August University Göttingen, Göttingen, Germany; 9 Department of Pathology, Georg-August University Göttingen, Göttingen, Germany; 10 Department of Urology, Caritas Clinics St. Joseph, University Regensburg, Regensburg, Germany; 11 Urological Group Practice & Clinic Derouet/Pönicke/Becker, Boxberg Center, Neunkirchen, Germany; 12 Department of Urology, Jena University Hospital, Jena, Germany; 13 for the German Network of Renal Cell Tumors, Jena, Germany; University of British Columbia, Canada

## Abstract

Collecting duct carcinoma (CDC) is a rare renal neoplasm that is associated with poor prognosis due to its highly aggressive course and limited response to immuno- or chemotherapy. Histologically, CDC is defined as a subtype of renal cell carcinomas, but in some cases, it is difficult to differentiate from urothelial carcinomas (UC). Therefore the aim of this study was to determine genetic alterations of CDC in comparison to that of urothelial carcinomas of the upper urinary tract (UUT-UC) to clarify the histological origin of this rare tumor entity. Twenty-nine CDC samples were obtained from seven different German centers and compared with twenty-six urothelial carcinomas of the upper urinary tract. Comparative genomic hybridization (CGH) was used to investigate the genetic composition of patients’ tumors and allowed the detection of losses and gains of DNA copy numbers throughout the entire genome. The clinical data were correlated with CGH results. CGH analysis of CDC revealed DNA aberrations in many chromosomes. DNA losses were more frequently observed than gains, while high-level amplifications were not detected. The mean frequency of CDC chromosomal aberrations (4.9/case) was slightly lower than that in UUT-UC (5.4/case). Recurrent CDC DNA losses occurred at 8p (n=9/29), 16p (9/29), 1p (n=7/29) and 9p (n=7/29), and gains occurred in 13q (n=9/29). In contrast to CDC, the most frequently detected UUT-UC DNA aberration was a loss at 9q (n=13/26). DNA losses at 9q, 13q and 8q as well as gains at 8p showed significant variations in UUT-UC compared to CDC. There was no correlation between the patients’ clinical course and the presence or absence of these recurrent genetic alterations. CDCs are characterized by a different genetic pattern compared to UUT-UC. Regarding the published data on renal cell carcinoma, we conclude that CDC appears to be a unique entity among kidney carcinomas.

## Introduction

Collecting duct carcinoma (CDC), a rare tumor of the kidney that accounts for 1-3% of all renal neoplasms, originates from the epithelium of the collecting ducts and is associated with aggressive course and poor prognosis. CDC cases predominantly present with metastases at time of diagnosis. Approximately two thirds of all patients with CDC die within two years of diagnosis. The mean age of CDC patients is younger than that of patients with other renal tumors (mean age at diagnosis: 43 years), and the disease predominantly affects males (male-to-female ratio 2:1) [1-10]. 

In literature, only approximately 400 total CDC cases are described. Recently, three large studies, each including more than 50 patients with CDC, were published. Two studies used data from the SEER database and a European multicenter study to develop a disease-specific risk model using histopathological and clinical parameters [6-8]. Additional data were presented in a multi-institutional clinical study from Japan (n=81) [9] and a matched subgroup analysis of a European nephrectomy database (n=41) [10]. Despite this accumulating information about the clinical management of CDC and patient outcome, little is known about the molecular origin of this tumor, and few papers have reported experimental studies on CDC [11-13].

The diagnosis of poorly differentiated, high grade carcinomas involving the renal sinus region is often difficult and one of the major criteria for diagnosis of CDC is exclusion of urothelial carcinoma involving the upper tract (UUT-UC). In addition, there are many clinical similarities between UUT-UC and CDC. However, the differentiation between these two tumor entities is important for the treatment of CDC [14-17]. Regarding this diagnostic dilemma several papers have been published in the last years [14-16]. Histologically, CDC is defined as a subtype of renal cell carcinomas (RCCs) and the discrimination from RCC subtypes is mostly not a challenging procedure in standard pathology, but CDC differs in presentation, radiologic observations and prognosis from other RCC. Several studies report that CDC demonstrates insufficient response to immunotherapy, chemotherapeutics (e.g. gemcitabine/cisplatin) and tyrosine kinase inhibitors [5,18]. As a result, the following question arose: should uro-oncologists treat these tumors as renal carcinomas or as urothelial carcinomas? To clarify the histological origin of CDC, we sought to determine whether CDC has genetic alterations similar to UUT-UC or RCC. Additionally, we compared cytogenetic results to clinical data from patients with CDC.

## Patients and Methods

### Ethics statement

Patients were identified in a retrospective database from several hospitals and samples were obtained from archival paraffin embedded tumor blocks, stored at Departments of Pathology. The use of de-identified human FFPE tissues had been approved by the institutional review board (Ethics Committee of the Medical Association of Saarland, No 188/05) without the necessity of individual informed consent. 

### Patients

Histological specimens from all patients with CDCs, as identified by the kidney tumor databases of the participating clinics of The German Network on Renal Cell Tumors, were re-evaluated by two experienced uropathologists (A.H. and R.G.). Cases of transitional cell carcinoma of the renal pelvis, papillary RCC type 2, sarcomatoid RCC, and several unclassified RCCs were excluded. After this histopathological review, 29 cases were definitively identified as CDC originating from seven participating clinics: Goettingen (n=8), Dresden (n=5), Regensburg (n=5), Jena (n=4), Homburg (n=4), Erlangen (n=2) and Moenchengladbach (n=1). At time of diagnosis, seven patients had localized disease without clinically detectable metastasis, whereas 22 patients had progressive CDC with lymph node infiltration or distant metastases.

UUT-UC samples of 26 patients who underwent radical nephroureterectomy in the urological clinics of Jena and Regensburg were collected as control group. Only tumors confirmed by uropathologists as UUT-UC were included.

### Comparative genomic hybridization (CGH)

DNA was isolated after macrodissection from three 10 µm thick, formalin-fixed paraffin-embedded tissue sections using the MagneSil® Genomic, Fixed Tissue Systems according to the manufacturer’s protocol (Promega, Mannheim, Germany). The DNA concentration was photometrically quantified using a NanoDrop spectrophotometer. A 2.5 µl aliquot of 4 ng/µl DNA dilutions was used as the starting material for the amplification. Phi29 amplification was performed according to the Repli-G kit manufacturer's instructions (Qiagen, Hilden, Germany) using an incubation time of 16 h. Repli-G reactions were examined using a real-time-based intra-*Alu* PCR assay [19]. 

The labeling, hybridization, washing and detection steps were performed using established protocols [20]. After hybridization, slides were analyzed with a digital image analysis system (MetaSystems ISIS 5.2, Altlussheim, Germany) using an Olympus AX61 microscope equipped with a CCD camera (ProgRes MF, Jenoptik, Jena, Germany). 

CGH results were correlated with the clinical course of CDC patients. Data are presented as means and ranges or as absolute and relative frequencies. Cancer-specific survival (CSS) was estimated using the Kaplan-Meier method. Statistical analysis was performed using the SPSS 19 software package (Chicago, Illinois); all tests were two-sided. As this was an exploratory study and no adjustment for multiple testing was performed, p-values are descriptive only. Different survival groups were compared using the log-rank test, and p-values < 0.05 were considered to be statistically significant.

## Results

Clinical and histopathological features of the 29 CDC patients and 26 UUT-UC patients are listed in Table 1. The mean age of the CDC patients was 66.3 years (range, 43-87 years) and comparable to the mean age of the UUT-UC patients, which was 68.8 years (range, 28-88 years). Approximately two thirds of the CDC patients were male (65.5%), whereas the male-to-female ratio of UUT-UC patients was balanced (14 men/12 women). Of the CDC patients, 44.8% were pN+ and 72.4% were cM+ at time of surgery. Of all CDC tumors, 62.1% had tumor stage pT3, 79.3% were Grade 3, 13.8% were Grade 4 and 6.8% were Grade 2 as determined by histopathological analysis (Table 1). The mean follow-up time for the CDC patients was 25.42 months. Among the UUT-UC patients, 61.5% had pTa or pT1 tumors; 38.5% of tumors were defined as stages pT2 to pT4. Of the UUT-UC tumors, 15.3% were determined to be G1, 50% G2 and 34.4% G3 tumors after histopathological review.

**Table 1:** Characteristics and clinical data of patients with CDC and UUT-UC.

		**CDC**	(n=29)	(%)	**UUT-UC**	(n=26)	(%)
**Sex**	male		19	(65.5)	male	14	(53.8)
	female		10	(34.5)	female	12	(46.2)
**Age**	mean		66.3		mean	68.8	
	range		43-87		Range	22-88	
**pT stage**	pT1a		2	(6.9)	pTa	14	(53.8)
	pT1b		3	(10.3)	pT1	2	(7.7)
	pT2		2	(6.9)	pT2	6	(23.1)
	pT3a		13	(44.8)	pT3	4	(15.4)
	pT3b		5	(17.3)	pT4	-	-
	pT4		4	(13.8)			
**Grading**	G II		2	(6.9)	G1	4	(15.4)
	G III		23	(79.3)	G2	13	(50.0)
	G IV		4	(13.8)	G3	9	(34.6)

The CSS data comparing the clinical course of localized vs. progressive tumors were only available for CDC patients. Both CDC groups had comparable follow-up times but significantly different survival rates (p<0.05). The five-year CSS was 26.2% in the progressive group (n=22) but was 100.0% for patients with localized disease (n=7).

**Figure 1:**
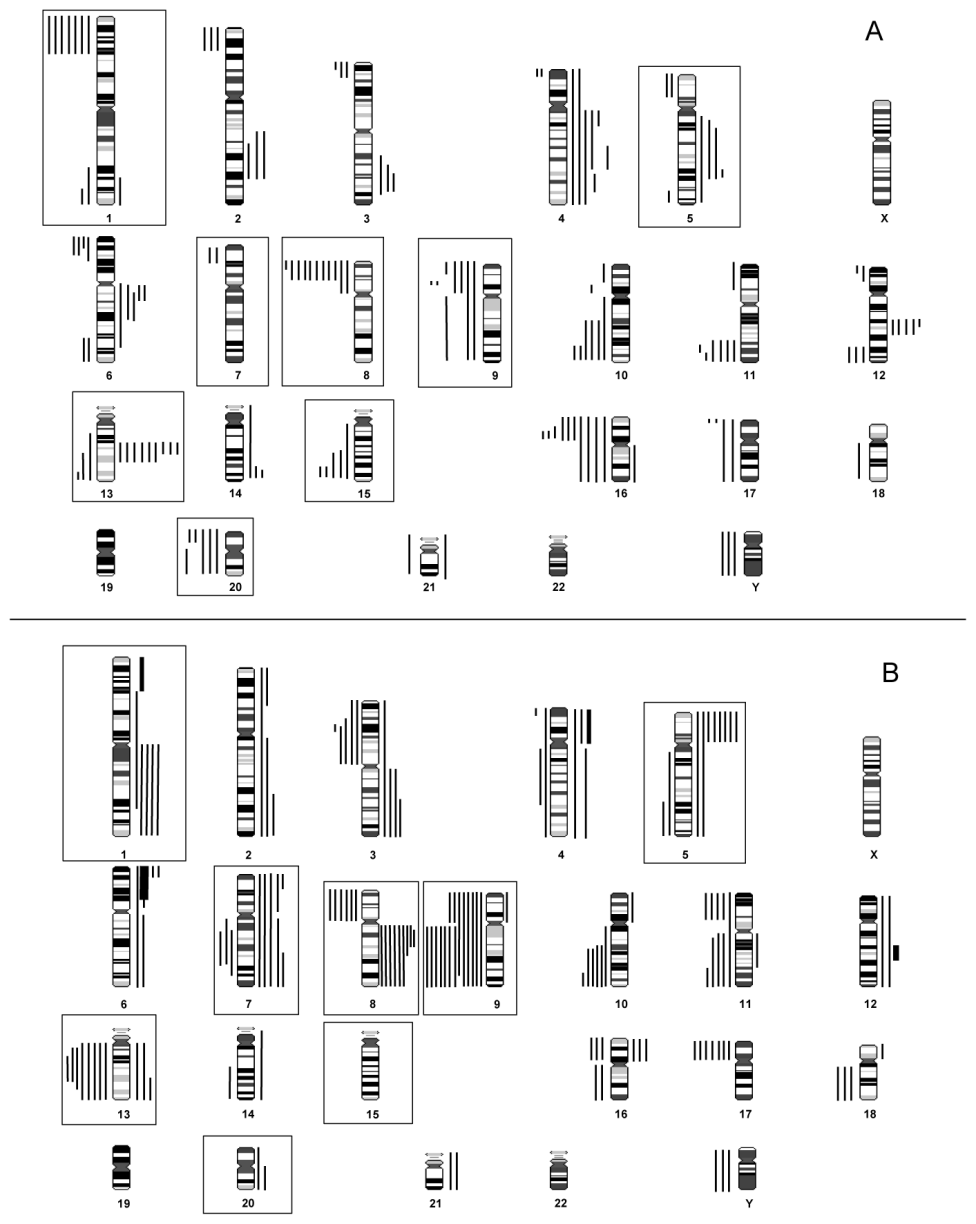
Superkaryogram of 29 CDC (A) and 26 UUT-UC (B) tumors. Losses of DNA are shown as lines on the left sides of chromosome ideograms, gains on the right side. High-level amplifications (amplicons) are shown as thick bars at the corresponding chromosome regions. Significant differences are highlighted by orange boxes.

CGH analysis results are summarized in Tables 2 and 3 as well as in Figure 1. CDCs showed chromosomal aberrations in different chromosomes; DNA losses were more frequently observed than gains. High-level gains of distinct chromosomal regions were not demonstrated (Figure 1a). Recurrent losses of chromosomal regions were detected on chromosomes 8p (n=9/29), 16p (n=9/29), 1p (n=7/29) and 9p (n=7/29), whereas recurrent gains were observed at 13q (n=9/29). Additionally, losses on chromosomes 10q (n=6/29), 11q (n=6/29), 20p (n=6/29), 15q (n=5/29), 20q (n=5/29), 6p (n=4/29) and 17p (n=4/29) and gains on chromosomes 4q (n=6/29), 6q (n=5/29), 12q (n=5/29) and 5q (n=4/29) occurred at lower frequencies. The observed mean overall frequency of chromosomal aberrations was 4.9/case in CDC patients (pT1 and pT2: 4.1/case; pT3 and pT4: 5.1/case). 

**Table 2:** CGH results of Collecting duct carcinomas (CDC): in detail.

**CDC T1-T2**								
**case**	**T Stage**	**losses**			**gains**	**Total losses**	**Total gains**	**Total defects**
DB3463II	1a	Y				1	0	1
DB01/11451	1a	10q24q26,16p13,20p13			6q11q14	3	1	4
DB00/23723	1b	10q24q26, 11q23q25, 12q24, 14q31q32, 15q24q26,16,17p13			2q21q32, 4q, 5q14q31, 6q11q16, 12q15, 13q14q21	7	6	13
U6437/03	1b	9			3q24,13q22q24	1	2	3
DB08/24085	1b	8p21p23,12q24,16p11p12,20q12q13			4q12q28, 5q13q21	4	2	6
25806 C2	2	3q32q34			-	1	0	1
P16229/08	2	9p			-	1	0	1
**CDC T3-T4**								
DB08/28952	3a	10p11p12,20			12q14q21,13q14q21	2	2	4
H12747	3a	1p36, 1q42q44, 2p23p25, 4p16, 5p15, 6p22p25, 8p22p23, 11p15, 11q25, 12p13, 13q34, 14q32, 15q,16p,17p13, 21			6q11q14	16	1	17
H15127	3a	8p, 10q			1q41q44	2	1	3
18603-08	3a	8p22p23, 9p21, 10q23q26			4q12q21, 13q21	3	2	5
P10834/00	3a	6p22p25, 6q25q27, 7p21, 8p21p23, 10q23q26,11q23q25			-	6	0	6
DB3812II	3a	1p36, 9q,11q23q25,Y			-	4	0	4
DB2144II	3a	8p21p23			12q15q21	1	1	2
9633 4 SS	3a	1p36			-	1	0	1
11510 A4	3a	9p21			-	1	0	1
U2581/04	3a	2p23p25			4, 6q13q21, 13q22q24	1	3	4
P8210/04	3a	8p23,15q25q26,16p21			3q21	3	1	4
P7118/07	3a	1p36, 8p21p23,11q25, 16, 18q			3q26	5	1	6
DB366II	3b	1p36,3p24p26,Y			13q14q21	3	1	4
9540A4	3b	-			-	0	0	0
12022B5	3b	8p22p23			5q23	1	1	2
DB27511-09	3b	5q34q35,9			-	2	0	2
DB07/32416	3b	8p21p23,9p,12q24,16,17,20			2q21q32, 3q21q27,6q11q23, 12q13q22	6	4	10
U7443/00	4	15q25q26			2q23q31, 4q26q28 ,5q21q22, 13q21	1	4	5
H15181IF	4	3p24p26, 6p24p25, 13q14q21,20p12p13			16q12q13	4	1	5
U867/04	4	15q23q26,16,20			4, 12q15, 13q21q31	3	3	6
DB29902	4	1p,16p			-	2	0	2
H15028-95	4	1p36,1q32q44, 2p23p25, 3p25p26, 4p16, 5p15, 6p22p25, 6q25q27, 7p21, 8p21p23, 9p24, 10p13p15, 10q23q26, 11q23q25, 12p12p13, 14,16p, 17, 20			-	19	0	19

**Table 3:** CGH results of Upper urothelial tract-urothelial carcinomas (UUT-UC) in detail.

**UUT-UC Ta-T1**										
**case**	**T Stage**	**Losses**	**Gains**		**Total losses**		**Total gains**		**Total defects**	
3322-90	Ta	11q22qter, 18q,Y	3q24qter, 5p, 8q, 12		3		4		7	
3902049	Ta	9	1q		1		1		2	
3473943	Ta	9q	7,13		1		2		3	
13724-96	Ta	9q, 10q25qter	-		2		0		2	
2727001	Ta	9q, 14q21qter	-		2		0		2	
3914353	Ta	3p21	-		1		0		1	
3384496	Ta	9,13q,17p	8q		3		1		4	
3689716	Ta	9q	4, 7p21pter		1		2		3	
3849366	Ta	5q31qter, 8p, 16q, 13q11q21	1q, 3, 5p, 6p, **6p21p25**, 7q22qter, 16p, 18p, 21		4		8		12	
5015410	Ta	16p, 17p	4q, 6q		2		2		4	
2395927	Ta	7q22q31, 9 10q23qter,11p,17p	-		5		0		5	
3834847	Ta	9	-		1		0		1	
2415-92	Ta	9	-		1		0		1	
3046251	Ta	9q, 11p	7, 13		2		2		4	
7940-98	1	8p, 9q, 11p	2q, 8q		3		2		5	
19495-95	1	8p, 11q, 16q	4p, 16p, 20q		3		3		6	
**UUT-UC ≥T2**										
7553-98	2	-	5, 8q		0		2		2	
8486-98	2	-	1q		0		1		1	
3719887	3	18q	6p22pter, 8q13q22		1		2		3	
6832-93	3	3p, 4p16pter, 9p, 10q23qter, 11q14qter, 13q, 17p, 18q, Y	1q,2,3q,5p,7q,12,14		9		7		16	
4990-96	3	8p,11p,13q,16p,17p	1p32q32,3q,**3q24qter**,7p,8q13q23,11q11q21,21		5		6		11	
2886206	3	13	2p16pter,5p,7,8q,10p,16p		1		6		7	
3968480	3	3p12p22,8p,10q21qter	-		3		0		3	
2505883	3	3p12p23,7q11q33,8p,9p,10q21qter,13q11q32,16p	2q32qter,4p,5p,6,8q		7		5		12	
1510534	3	3p, 4, 5q13qter, 9q, 10q, 11,13q11q31, 17p, 21	**1p32pter**, 5p, 6p22pter, **8q21**, 9p, **12q12q15**, 13q31qter, 20		9		8		17	
18899-96	3	4q12q31, 7q21q34, 9pterq32, 11q, 13, 17p, Y	-		7		0		7	

Amplicons are depicted in bold type

In comparison to the CDC tumor samples, the mean chromosomal aberration frequency in UUT-UC tumors was similar (5.4/case), whereas 3.9 and 7.9 aberrations were found in pTa/pT1 and ≥pT2 stage tumors. Amplification of chromosomal regions on 1p, 3q, 6p, 8q and 12q was observed in three different cases (Figure 1b), whereas losses on 9q were found in 50% of cases. Further recurrent losses were detected on chromosomes 11 (n=9/26), 13 (n=7/26), 9p (7/26), 17 (n=7/26), 10 (n=6/29) and 3, 8p and 16 (n=5/26). Frequent gains were observed in chromosomal regions 8q (n=9/26), 7 (n=7/26), 1 (n=6/26), 5 (n=6/26) and 6 (n=5/26).

**Figure 2:**
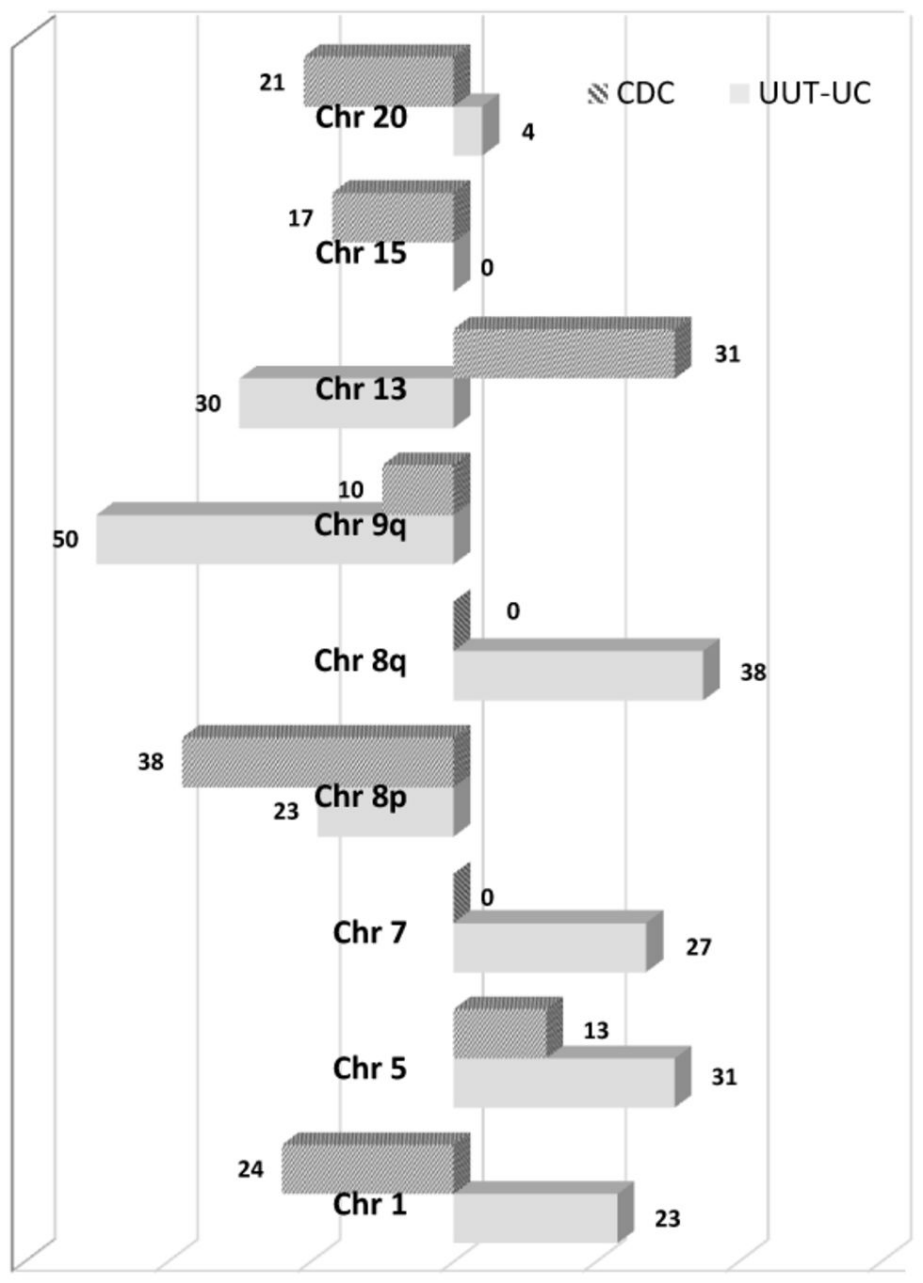
Comparison of chromosomal aberrations in CDC and UUT-UC tumors in percent. Losses of chromosomal material are shown on the left side, gains on the right side.

Based on our CGH data, CDC and UUT-UC demonstrated a similar average number of chromosomal aberrations. However, differences between CDC and UUT-UC can be observed in many chromosomal regions. Amplifications of distinct chromosomal regions occurred only in UUT-UC. Significant differences involved losses of 1p, 8p, 9q, 13q, 15q, 20 as well as gains on 5p, 6p24, 7 and 8q (Figure 2).

Next, we compared the differences in genomic imbalances between organ confined and advanced stages (Table 4). Except for the loss on chromosome 9q, which was detected in 50% of <T2 stage tumors and only in 20 % of the advanced UUT-UC, we observed a higher number of chromosomal alterations in advanced stage tumors. In CDCs, the frequency of chromosomal imbalances in advanced-stage tumors was similar to organ-confined diseases. However, losses on 1p36, 3p and 6p as well as a gain on chromosome 13 were associated with aggressive disease stages. 

**Table 4:** Comparison of the most common aberrations and their frequencies in CDC and UUT-UC tumors.

**Recurrent gains**							
**CDC T1-T2 (n=7)**		**CDC T3-T4 (n=22)**		**UUT-UC Ta-T1 (n=16)**		**UUT-UC ≥T2 (n=10)**	
**Region**	**n (%)**	**Region**	**n (%)**	**Region**	**n (%)**	**Region**	**n (%)**
1q	-	1q	1 (4.5)	1q	2 (12.5)	1q	2 (20)
2q	1 (14.2)	2q	2 (9.0)	2q	1 (6.25)	2q	2 (20)
3q	1 (14.2)	3q	3 (13.5)	3q	2 (12.5)	3q	2 (20)
5p	-	5p	1 (4.5)	5p	2 (12.5)	5p	5 (50)
6p24	-	6p24-ter	-	6p24-ter	6 (37.5)	6p24-ter	3 (30)
7	-	7	-	7	4 (25.0)	7	1 (10)
8q	-	8q	-	8q	3 (18.75)	8q21	6 (60)
12q	2 (28.4)	12q	5 (22.5)	12q	1 (6.25)	12q	2 (20)
13q22-24	1 (14.2)	13q22-24	5 (22.5)	13q22-24	2 (12.5)	13q22-24	1 (10)
**Recurrent losses**							
**CDC T1-T2 (n=7)**		**CDC T3-T4 (n=22)**		**UUT-UC Ta-T1 (n=16)**		**UUT-UC ≥T2 (n=10)**	
**Region**	**n (%)**	**Region**	**n (%)**	**Region**	**n (%)**	**Region**	**n (%)**
1p36	-	1p36	7 (31.8)	1p36	-	1p36	-
3p	-	3p	3 (13.5)	3p	1 (6.25)	3p	4 (40)
6p24-25	-	6p24-25	4 (18.0)	6p24-25	-	6p24-25	-
8p	1 (14.2)	8p	10(45.5)	8p	3 (18.75)	8p	3 (30)
9p	2 (28.4)	9p	5 (22.5)	9p	5 (31.25)	9p	3 (30)
9q	1 (14.2)	9q	3 (13.5)	9q	11 (68.75)	9q	2 (20)
10q23-ter	2 (28.4)	10q23-ter	3 (13.5)	10q23-ter	1 (6.25)	10q23-ter	4 (40)
13q11-31	-	13q11-31	3 (13.5)	13q11-31	1 (6.25)	13q11-31	5 (50)
15q25-ter	1 (14.2)	15q25-ter	4 (18.0)	15q25-ter	-	15q25-ter	-
16p12	2 (28.4)	16p12	7 (31,8)	16p12	1 (6.25)	16p12	2 (20)
17p	1 (14.2)	17p	3 (13.5)	17p	3 (18.75)	17p	4 (40)
20	2 (28.4)	20	5 (22.5)	20	-	20	-

No correlations between the genetic pattern, different clinical appearances and courses of disease in CDC were detected. Patients with unfavorable outcomes (progressive CDC) had no specific or recurrent alterations compared to patients with localized tumors. These findings were also confirmed as not significant compared with pN0 and pN+ or pM0 and pM+ patients. 

## Discussion

The CDC cases reported in this nationwide multi-center study represent one of the largest series combining molecular genetic experimental and clinical findings. Until 2002, only 40 cases of CDC worldwide have been summarized in a review paper; most cases were described by case reports [17]. In 2006, a nationwide survey in Japan characterized 80 cases of CDC [9]. The European multi-institutional clinical study described CSS of 41 CDC cases matched with RCC-cases [10]. In contrast to the Japanese group (five-year CSS of 34.3%), here, similar CSSs for both tumor types (5-year CSS of 48% for CDC vs. 57% for RCC) have been demonstrated [10]. Another paper reported an OS of 17% in 23 patients with metastatic disease [9]. Recently, three large studies were published that evaluated the prognostic value of clinical and histopathological parameters for clinical decision making in patients with CDC. Two American population-based studies used surveillance and epidemiological data from the SEER database of CDC patients and compared the survival rates to ccRCC (n=160) [6] or medullary renal cell carcinoma (n=227) [7]. A five-year CSS was not determined in either study, but Wright et al [6] estimated a three-year CSS of 58%. Recently, a European multi-center study with 95 CDC patients collected from 16 different centers reported a five-year CSS of 40.3% with a median follow-up time of 48.1 months [8]. Our five-year CSS of 38.4% is similar to the reported data. In the present study, there is most likely a subgroup of patients included with localized tumors and an accordingly favorable outcome. This result supports the data from the European study, which evaluated a subset of low-risk patients with excellent survival based on different histopathological and clinical variables in more than a quarter of the patients (disease specific mortality of 4 % after five years) [8]. The prognostic relevance was determined by establishing a disease-specific risk model that considered tumor size, metastasis, grade, and lymph vessel infiltration [8]. To date, no reports have documented that patients with localized CDC who are treated by nephron-sparing renal surgery or radical nephrectomy have favorable outcomes and that as a result, surgery could be a curative treatment option [21,22]. Unfortunately, the number of localized CDCs in the present study is small (n=7), and the mean follow-up is rather short (28.3 months), but a 100% CSS of two patients after five years has been calculated.

Our experience with CDC indicates that many potential CDCs collected in retrospective databases would be reclassified as other aggressive tumor types after histopathological re-evaluation. Hence, only a small proportion of these potential CDCs could be verified as CDC by experienced uropathologists. In all retrospective clinical studies concerning CDC, with the exception of the nationwide Japanese survey [9], no additional reviews by an experienced pathologist were performed [6-10]. 

Knowledge of the molecular basis of CDC is still limited. Only few inconsistent experimental studies are available, and the potential use of these studies for molecular classification is hampered by the facts that most studies were based on small groups and that different techniques were used for molecular characterization [11-13,23-25]. Cytogenetic analysis has shown that CDCs are characterized by genetic losses in chromosomes 1, 6, 8, 14, and 15 and gains in chromosomes 16 and 20 [2,11,13,24]. In the present study, several of these alterations were confirmed, but we additionally detected new losses on chromosomes 1, 9, 16 and 20 in at least 20 % of the analyzed cases. Gains on chromosomes 16 and 20 were not detected, but gains on chromosome 13 and X were observed. Chromosomal aberrations associated with RCC and UC of the renal pelvis have not been reported [26-29]. Additionally, Schoenberg et al described an LOH in 8p and 13q in 50% of the 6 cases analyzed. However, no LOH on 3p was detected, which also differentiates CDC from RCC [12]. Accordingly, we detected a 3p loss - a typical alteration of clear cell RCC - in only 3 cases. This observation is confirmed by the results of other cytogenetic and FISH studies [11,23,24]. In addition, often described specific chromosomal imbalances of clear cell RCC tumors like loss of 6q and 14 and gains of 5, 7 and 12 were infrequent findings in CDC tumors. Moreover we did not find in CDC any characteristic aberrations associated with papillary RCC like gains of 7, 16 and 20 and in only 13% of CDC a loss of 17p was detected, which suggests that CDC represents a unique renal tumor entity [26,27,30]. The comparison to the genetic composition of RCC was performed with data published in the literature. Cytogenetic alterations of RCC and its different subgroups are well documented and generally accepted in many studies published during the last years [31-33]. Therefore we used this data for the differentiation of CDC to RCC because we believe, that the use of own experimental findings would not differ from the known data and would not give more information for discrimination of CDC and RCC on the genetic level.

Another important point is the differentiation of CDC from urothelial carcinomas. Orsola et al reported similar characteristics in CDC and UUT-UC by immunostaining [14]. However, we could clearly differentiate both tumor entities at the cytogenetic level. UUT-UC tumors are relatively rare and account for approximately 10% of all renal tumors. Although chromosomal abnormalities in bladder UC have been well documented, cytogenetic studies of UUT-UC are rare. The cytogenetic profile of UUT-UC has been reported to be identical to that of bladder UC [28,29]. Frequent observations in bladder UC were chromosomal gains on 1q, 5p, 7p, 8q, 13q, 17q, 20q and losses on 3p, 5q, 8p, 9 and 11 [29]. When compared to our results, many of the observed genetic changes were consistent to UUT-UC alterations except the gains on 17q and 20q as well as losses on 5q and 11. In contrast to a previously published array-CGH analysis of 32 UUT-UC patients, we observed a higher mean frequency of chromosomal alterations in ≥T2 vs. Ta-T1 stage tumors (7.9 vs. 3.9/case) [34]. Interestingly, we detected losses of 9q in 50% of pTa/T1 tumors but only in 20% of invasive stage, whereas gains on 8q and losses of 10q and 13q were more frequently observed in advanced stage. This observation underlines the assumption that distinct genetic alterations might be initial events in the tumorigenesis of low grade and high grade UUT-UC.

In summary, comparison of the CDC genetic composition with that of other renal tumor entities revealed no correlation with RCC or UUT-UC [7,26-30]. Unfortunately, no associations between clinical characteristics and cytogenetic findings have been detected. 

Although CGH has proven to be a useful technique to give an overview of the chromosomal composition of tumors, the application is affected with some limitations. This reflects the limited resolution of aberrations smaller than 5–10 Mb which cannot be detected and the inability to detect structural chromosomal alterations, such translocation, inversion or other complex rearrangements. Other limitations of the presented study are the retrospective setting; the small group of CDC patients, especially those with localized tumors; and the relatively short follow-up time. Only a large, worldwide, prospectively designed registry of CDC could accumulate an adequate number of patients to confirm our hypotheses. Further investigations with the existing registry and newly collected cases from additional centers are planned to reveal CDC-associated pathways and the molecular basis of this disease as well as additional characteristics of this rare renal tumor entity.

## Conclusions

For the first time, we have shown that CDC is characterized by a different genetic pattern compared to UUT-UC. CDC therefore appears to be a unique kidney tumor based on the genetic profile compared to RCC and UUT-UC.

These genetic alterations did not show any correlations with the patients’ clinical course. However, the clinical data differed from those described in previous publications. Patients with lymph node involvement or metastases at the time of diagnosis are characterized by an unfavorable clinical course. In contrast, patients with localized CDC have a five-year CSS of 100%. Furthermore, multi-institutional investigations of CDC using a larger number of patients are necessary to confirm these preliminary results and to understand the molecular mechanisms of this rare tumor.
